# Supplementary material for: Demographic profiles of contact lens wearers and their association with lens wear characteristics in Trinidad and Tobago: A retrospective study
Source: PLoS One. 2022 Jul 22;17(7):e0264659. doi: 10.1371/journal.pone.0264659 (PMC9307171; doi:10.1371/journal.pone.0264659)
Supplement: Attachment — Submitted filename: Rubbal letter.docx [file pone.0264659.s002.docx]

**Response to reviewers’ comments**

Thank you for the very useful comments. We have revised the manuscript according to the constructive comments made by the reviewers. Please find below a point-by-point response to the reviewers’ comments.

**Reviewer 1**

1. **Reviewers’ comment**

Line 145: The authors should define the meaning of the different purpose of wear, particularly fashion and therapeutic/cosmesis.

**Response**

Done. The meanings of fashion and therapeutic/cosmesis have been defined in the manuscript (line 144 to 147).

2. **Reviewers’ comment**

Table 1 and Line 171. Cosmesis is not report in the table 1. The authors should always use the same terminology to make the reading of the work simpler and avoid confusing the reader.

**Response**

Cosmesis and fashion are basically the same thing in this contest. To avoid confusion, we have used fashion as it best represents the responses. This has been corrected in the table and the entire manuscript as suggested.

3. **Reviewers’ comment**

Line 176-177:

“Most (61%) record cards did not reflect the type of lens system used.” Insert lens care system.

**Response**

Lens care system has been inserted as suggested (line 179)

4. **Reviewers’ comments**

The authors should define which type of lens care is “Boston solution” because Boston solution is a brand of Bausch and Lomb Company which includes many different types of solutions.

The authors should include the company data in the manuscript

**Response** The type of Boston solution used has been defined in the manuscript (line 181 to 182). It now reads:

**‘**Most (61%) record cards did not reflect the type of lens care system used. About one-quarter (24.3%) used multipurpose solution [e.gs include Bausch and Lomb Renu advance formula, Renu fresh, Renu sensitive and Biotrue multipurpose solutions used for soft CLs] and 14% used Boston solution **by** Boston Advance® care system (Bausch & Lomb, Rochester, New York)’. See also footnote in Table 1

5. **Reviewers’ comment**

Lines 92 and 95: Trinidad y Tobago had been previously defined; authors should use the abbreviations.

**Response**

Trinidad and Tobago have been changed to T & T in the entire manuscript as suggested

**Reviewers’ #2**

6. **Reviewers’ comment:** As stated in the methods section the study is a retrospective study that reviewed Cl wearers’ clinical records of 243 CL wearers attending a university clinic. However, in the abstract authors should not refer to the record as “About half of the respondents wear”. as there is now acquired responses from the wearers. (lines 40-41).

**Response**

The statement has been corrected to read’’ About half of the CL wearers used them for fashion (more among those aged 18 to 30 years, 61.0%), therapeutic (more among those <18 years, 43.8%, *P* = 0.001) and refractive error correction purposes (more in those >40 years, *P* =0.001)’’ (line 45 to 47)

We have also replaced “respondents” with “CL wearers” in the manuscript as suggested. In the abstract (method section) we have also clearly stated that the study was retrospective.

7. **Reviewers’ comment**

Lines 140-141

Exclusion criteria, why to exclude contact lens prescribed outside UWI optometry clinic. As the aim of the study is to study characteristic of contact lenses in the population rather than single clinic.

**Response**

This was a retrospective study involving records of patients that attended the UWI optometry clinic. This was also made clear in the abstract (method section). For this reason, those who CLs were not prescribed at the UWI clinic were excluded because the information regarding their CLs and other variables needed in this study cannot be verified.

8. **Reviewers’ comment**

The author mentioned a brand of Boston solution, can more details add is it RGP solution? ANY OTHER DETAILS.

**Response**

More details about the type of Boston lens care solution used have been added. The section now reads:

“Boston solution by Boston Advance® care system (Bausch & Lomb, Rochester, New York) (line 180 to 181)”. See also footnote in Table 1

9. **Reviewers’ comment**

Table 1 for better readability and comparison to include number of participants and percentage in one column n (%).

**Response**

The number of participants and percentage have been put in one column as suggested (see Table 1) and footnote added.

10. **Reviewers’ comment**

Table 1 CL type the sum of percentages does not give a total of 100%

**Response**

The percentage has been corrected.

11. **Reviewers’ comment**

The discussion could include more elaboration on the findings mentioned in the result section,

Age, gender, and employment status

**Response**

The discussion has been elaborated to include age, gender, and employment status in the manuscript (line 240 to 271).

12. **Reviewers’ comment**

Line 263 “questionnaire-based studies”, the study is retrospective no mention of any questionnaire or patients’ interview.

**Response**

Retrospective studies include review of patient record as well and does not always have to be surveys or interviews. This has been reflected more across the manuscript. Some of the relevant sections were:

“This was a retrospective review of data from patients prescribed with CL” (Line 134)

And

This retrospective study investigated the characteristics of CL wearers and their association with lens characteristics using data (line 234 to 235).

13. **Reviewers’ comment**

Conclusion: respondent word should not be used in the study as it is retrospective based

**Response**

We have replaced “respondents” with “CL wearers” in the manuscript as suggested and have also rephrased the conclusion.
